# Prolonged overall treatment time negatively affects the outcomes of stereotactic body radiotherapy for early-stage non-small-cell lung cancer: A propensity score-weighted, single-center analysis

**DOI:** 10.1371/journal.pone.0253203

**Published:** 2021-06-18

**Authors:** Toshiki Ikawa, Takahiro Tabuchi, Koji Konishi, Masahiro Morimoto, Takero Hirata, Naoyuki Kanayama, Kentaro Wada, Masayasu Toratani, Sumiyo Okawa, Kazuhiko Ogawa, Teruki Teshima

**Affiliations:** 1 Department of Radiation Oncology, Osaka International Cancer Institute, Osaka, Japan; 2 Cancer Control Center, Osaka International Cancer Institute, Osaka, Japan; 3 Department of Radiation Oncology, Osaka University Graduate School of Medicine, Osaka, Japan; St. Vincent Medical Center, UNITED STATES

## Abstract

Previous studies have reported conflicting results for the effect of overall treatment time with stereotactic body radiotherapy on tumor control in early-stage non-small-cell lung cancer. To examine this effect, we conducted a propensity score-weighted, retrospective, observational study at a single institution. We analyzed the data of 200 patients with early-stage non-small-cell lung cancer who underwent stereotactic body radiotherapy (48 Gy in 4 fractions) at our institution between January 2007 and October 2013. Patients were grouped into consecutive (overall treatment time = 4–5 days, n = 116) or non-consecutive treatment groups (overall treatment time = 6–10 days, n = 84). The outcomes of interest were local control and overall survival. The Cox regression model was used with propensity score and inverse probability of treatment weighting. The median overall treatment times in the consecutive and non-consecutive groups were 4 and 6 days, respectively. The 5-year local control and overall survival rates in the consecutive *vs*. the non-consecutive group were 86.3 *vs*. 77.2% and 55.5 *vs*. 51.8%, respectively. After propensity score weighting, consecutive stereotactic body radiotherapy was associated with positive local control (adjusted hazard ratio 0.30, 95% confidence interval 0.14–0.65; p = 0.002) and overall survival (adjusted hazard ratio 0.56, 95% confidence interval 0.34–0.91; p = 0.019) benefits. The prolonged overall treatment time of stereotactic body radiotherapy treatment negatively affected the outcomes of patients with early-stage non-small-cell lung cancer. To our knowledge, this is the first study to show that in patients with early-stage non-small-cell lung cancer treated with the same dose-fractionation regimen, consecutive stereotactic body radiotherapy has a more beneficial effect on tumor control than non-consecutive stereotactic body radiotherapy.

## Introduction

Stereotactic body radiotherapy (SBRT) or stereotactic ablative radiotherapy allows accurate delivery of very high radiation doses in a small number of fractions at appropriate target volumes using highly conformal radiotherapy planning, respiratory motion management, and daily image guidance [1, 2]. Currently, SBRT is the primary treatment modality with excellent local control (LC) and low toxicity rates in patients with inoperable early-stage non-small-cell lung cancer (NSCLC) and in patients who refuse surgery [2–5].

The optimal dose-fractionation schedule of SBRT for early-stage NSCLC to maximize tumor control and minimize toxicity has not been determined. The dose-fractionation schedule comprises the total dose, number of fractions, and overall treatment time (OTT). Several studies have shown that LC was better in patients treated with a biologically effective dose (BED) using an α/β ratio = 10 Gy (BED10) of at least 100–105 Gy than in patients treated with less than 100–105 Gy [6–9]; BED estimates the biological effects of different dose-fractionation schemes on both tumors and normal tissues [10]. Conversely, few studies have reported the influence of OTT on SBRT outcomes in early-stage NSCLC, but their results were inconsistent [6, 11–13]. For example, Kestin *et al*. [6] demonstrated that a shorter treatment time (≤ 10 days) was associated with a better LC in a retrospective study including 483 patients who underwent SBRT for NSCLC at five institutions. In contrast, Samson *et al*. [11] retrospectively analyzed the data of 245 patients from two facilities who were treated with SBRT for cT1–2 NSCLC but observed no associations between the treatment schedule (≤ 7 *vs*. > 7 days) and LC and overall survival (OS). However, these studies included two or more dose-fractionation regimens, which may have affected the outcomes. To overcome this limitation, we need to compare the influence of OTT on tumor control in patients treated with the same dose-fractionation regimen. Therefore, we conducted a retrospective, observational study in patients with early-stage NSCLC who underwent SBRT with the same dose-fractionation regimen.

## Materials and methods

### Patients

We identified 222 patients who received SBRT for primary lung cancer at our institution between January 2007 and October 2013. The inclusion criteria were (1) the presence of newly diagnosed primary NSCLC with or without biopsy confirmation, regardless of prior treatment for lung cancer; (2) the presence of clinical stage T1–3N0M0 cancer (based on International Union Against Cancer Tumor, Node, Metastasis classification, 7^th^ edition); (3) the presence of a tumor of diameter less than 5 cm; (4) the receipt of SBRT of 48 Gy in 4 fractions; and (5) follow-up for at least 6 months. The exclusion criteria were patients with cytologically or histologically diagnosed small-cell lung cancer and those who received other dose fractionation regimens or adjuvant chemotherapy. Twenty-two patients were excluded, and 200 patients who met our inclusion criteria were enrolled.

This retrospective study was approved by the ethics committee of Osaka International Cancer Institute (approval number 19164). All patients provided written informed consent for the use of their data in clinical research before the administration of SBRT and had the opportunity to opt-out of the study.

### Treatment procedures

All patients were immobilized with the BodyFix double-vacuum immobilization system (Medical Intelligence, Schwabmuenchen, Germany) and observed using four-dimensional computed tomography (CT). The internal target volume was determined using four-dimensional CT images encompassing the gross tumor volumes in all respiratory phases. The planning target volume was generated by expanding the internal target volume by 5–8 mm in all directions. A total dose of 48 Gy in 4 fractions was prescribed at the isocenter using 6–9 non-coplanar static conformal beams with a 5 mm multi-leaf collimator margin. All treatment plans were generated using the Eclipse treatment planning system (Varian Medical Systems, Palo Alto, CA, USA). The radiation doses before January 2009 were calculated using a pencil beam algorithm with an inhomogeneity correction and thereafter with an analytical anisotropic algorithm. Image guidance before September 2007 was based on bony anatomy matched with orthogonal kV imaging and thereafter, based on tumor matching with online three-dimensional cone-beam CT imaging. All treatments were delivered using a linear accelerator (Clinac 23EX, Varian Medical Systems, Palo Alto, CA, USA).

### Patient follow-up and evaluation of local failure

Follow-up examinations after SBRT typically consisted of a chest CT every 3 months for 1 year, and thereafter, every 6 months. When relapse was suspected, ^18^F-fluorodeoxyglucose-positron emission tomography-CT (^18^F-FDG-PET/CT) was performed. Local failure was defined as recurrence within 1 cm of the planning target volume as described by the Radiation Therapy Oncology Group 0236 trial [3] and confirmed using biopsy of the viable carcinoma or the accumulation of fluorodeoxyglucose to maximum standardized uptake value (SUVmax) ≥ 5.0 or pretreatment SUVmax [14]. When these examinations were not feasible, local failure was diagnosed based on the presence of high-risk CT features as proposed by Huang *et al*. [15] In patients who were not followed up in person, local failure was evaluated based on information from their new physicians.

### Outcomes

We grouped patients into consecutive (OTT = 4 or 5 days, n = 116) and non-consecutive treatment groups (OTT = 6–10 days, n = 84) (i.e., in the consecutive group, SBRT was administered within a calendar week, whereas, in the non-consecutive group, SBRT was administered over two calendar weeks with a treatment-break because of the weekend). The primary and secondary outcomes of interest were LC and OS, respectively, across the two groups. We performed all analyses at the patient level and restricted the follow-up period to the first 5 years after treatment. LC was defined as the time from the start of SBRT to the date of local failure. OS was defined as the time from the start of SBRT to the date of death from any cause.

### Statistical analyses

The differences in baseline characteristics between the two groups were assessed using the Fisher’s exact test or chi-squared test. To account for the imbalance of baseline covariates between the two groups, we performed a propensity score (PS) analysis using the inverse probability of treatment weighting. PS values were estimated from covariates using multivariable logistic regression and plotted as histograms. Model discrimination was assessed using the receiver operating characteristic curve and concordance statistic (c-statistic). Covariate balance was assessed using a standardized mean difference approach. A standardized mean difference of less than 0.10 for a given covariate was considered as an acceptable balance. Unadjusted LC and OS curves were shown using the Kaplan–Meier method and compared using the log-rank test, and the corresponding hazard ratios (HRs) were estimated. Adjusted LC and OS curves were shown using the Cox proportional hazards model with PS-weighting, and the adjusted HRs were estimated. Furthermore, to account for the residual covariate difference after PS-weighting, direct covariates-adjusted survival curves [16] with PS-weighting were generated using the covariates that we used to estimate PS, and the corresponding adjusted HRs were estimated. To validate the robustness of the results, we performed a sensitivity analysis comparing 4 days of consecutive treatment with 6–10 days of non-consecutive treatment (excluding patients treated with an OTT of 5 days) and conducted multivariable Cox proportional hazards analyses including the following three OTT subgroups: 4–5 days (n = 116), 6 days (n = 56), and 7–10 days (n = 28).

All statistical tests were two-sided, and a p-value of less than 0.05 was considered statistically significant. All analyses were performed using R version 3.5.3 (R Foundation for Statistical Computing, Vienna, Austria) and SAS version 9.4 (SAS Institute Inc., Cary, NC, USA).

## Results

### Patient characteristics and propensity score weighting

**Fig 1:**
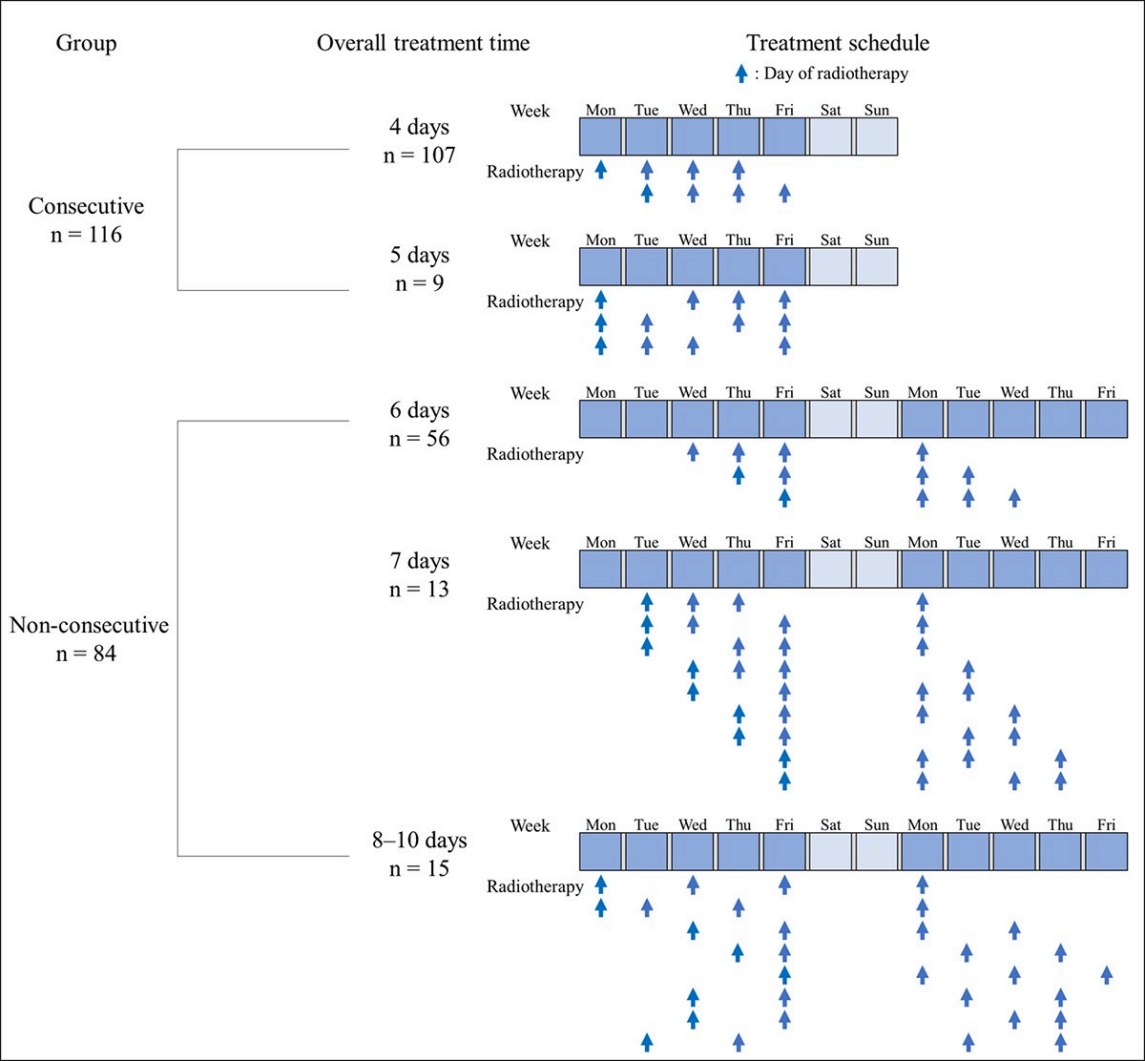
Graphical depiction of the stereotactic body radiotherapy schedules for the consecutive and non-consecutive treatment groups.

The typical treatment schedules for each group are shown in Fig 1. In the consecutive group, treatment breaks of 1 day occurred in 9 patients because of public holidays or machine maintenance. In the non-consecutive group, treatment breaks of 2–6 days occurred in 73 patients because of weekends, public holidays, or machine maintenance, in 9 patients because of every-other-day SBRT based on the treatment protocol, and in 2 patients because of personal reasons. The median OTT in the consecutive group was 4 (range = 4–5) days, and approximately 90% (n = 107) of the patients received SBRT over 4 days. In the non-consecutive group, the median OTT was 6 (range = 6–10) days, and approximately 70% (n = 56) of the patients received SBRT over 6 days (S1 Fig).

Baseline characteristics of eligible patients, stratified based on consecutive *vs*. non-consecutive treatment, are shown in Table 1. A significant difference was observed in the calendar period of treatment (p <0.001). The use of non-consecutive SBRT was more frequent in 2009–2010 and 2011–2013 than in 2007–2008 (p <0.001 and p = 0.005, respectively) because during 2007–2008, we conducted SBRT more frequently at the hospital than at an outpatient setting, and thus we tended to initiate SBRT at the beginning of a weekday. The standardized mean differences of unweighted comparisons significantly differed in all covariates except for tumor location (Table 1). The distributions of the estimated PS, receiver operating characteristic curve, and c-statistic are shown in S2 Fig. After PS weighting, the standardized mean difference for all covariates was confirmed to be less than 0.10, which indicated that the distribution of all covariates was adequately balanced (Table 1).

**Table 1:** Patient characteristics in the two treatment groups and standardized mean differences before and after propensity score weighting.

	Unweighted, n (%)		p-value*	Propensity score-weighted, %		Standardized mean difference	
Characteristic	Consecutive n = 116	Non-consecutive n = 84		Consecutive	Non-consecutive	Unweighted	Propensity score-weighted
**Age (years)**			0.65			0.13	0.041
** 50–69**	14 (12.1)	11 (13.1)		12.6	11.6		
** 70–79**	46 (39.7)	38 (45.2)		41.1	40.2		
** 80–90**	56 (48.3)	35 (41.7)		46.3	48.2		
**Sex**			0.34			0.16	0.013
** Female**	29 (25.0)	27 (32.1)		27.1	27.7		
** Male**	87 (75.0)	57 (67.9)		72.9	72.3		
**Histological type**			0.72			0.12	0.089
** Adenocarcinoma**	51 (44.0)	38 (45.2)		44.9	41.4		
** Squamous cell carcinoma**	26 (22.4)	15 (17.9)		21.4	24.8		
** Non-biopsy-proven**	39 (33.6)	31 (36.9)		33.7	33.8		
**Tumor size**			0.28			0.23	0.030
** 1a (≤2 cm)**	63 (54.3)	38 (45.2)		48.8	48.0		
** 1b (>2 cm, ≤3 cm)**	32 (27.6)	32 (38.1)		33.0	32.7		
** 2a (>3 cm, ≤5 cm)**	21 (18.1)	14 (16.7)		18.1	19.3		
**Pretreatment SUVmax**			0.37			0.20	0.060
** ≤5**	61 (52.6)	52 (61.9)		56.4	54.2		
** >5**	47 (40.5)	26 (31.0)		36.9	39.8		
** Unknown**	8 (6.9)	6 (7.1)		6.7	6.0		
**Tumor location**			0.62			0.09	0.004
** Lower lobe**	35 (30.2)	29 (34.5)		33.0	32.9		
** Upper/Middle lobe**	81 (69.8)	55 (65.5)		67.0	67.1		
**Charlson comorbidity index [17]**			0.30			0.18	0.012
** 0–2**	90 (77.6)	71 (84.5)		80.7	80.2		
** >2**	26 (22.4)	13 (15.5)		19.3	19.8		
**Prior history of surgery or radiotherapy for lung cancer**			0.23			0.19	0.029
** No**	90 (77.6)	58 (69.0)		77.1	78.3		
** Yes**	26 (22.4)	26 (31.0)		22.9	21.7		
**Calendar period of treatment**			< 0.001			0.62	0.025
** 2007–2008**	50 (43.1)	14 (16.7)		32.0	33.1		
** 2009–2010**	32 (27.6)	40 (47.6)		37.2	36.3		
** 2011–2013**	34 (29.3)	30 (35.7)		30.9	30.6		
**History of smoking**			0.20			0.21	0.014
** No**	18 (15.5)	20 (23.8)		20.3	20.9		
** Yes**	98 (84.5)	64 (76.2)		79.7	79.1		

*p-values were calculated before propensity score weighting.

SUVmax, maximum standardized uptake value.

### Outcomes

The follow-up rates in the consecutive and non-consecutive groups were 88.8 and 86.9% at 3 years and 75.9 and 78.6% at 5 years, respectively. In the consecutive group, 13 (11.2%) patients experienced local failure and 44 (37.9%) patients died, whereas in the non-consecutive group, 16 (19.0%) patients experienced local failure and 35 (41.7%) patients died (Table 2). Of the 13 patients who experienced local failure in the consecutive group, 2, 10, and 1 were diagnosed based on biopsy, ^18^F-FDG-PET/CT, and CT findings, respectively. Of the 16 patients who experienced local failure in the non-consecutive group, 4 and 12 were diagnosed based on biopsy and ^18^F-FDG-PET/CT findings, respectively.

**Table 2:** Crude event rates of local failure and death according to the overall treatment time.

Overall treatment time (days)	Local failure events/n	%	Death events/n	%
**4**	11/107	10.3	40/107	37.4
**5**	2/9	22.2	4/9	44.4
**6**	10/56	17.9	24/56	42.9
**7**	2/13	15.4	3/13	23.1
**8**	2/9	22.2	5/9	55.6
**9**	0/2	0	1/2	50.0
**10**	2/4	50.0	2/4	50.0
**Consecutive (4 or 5 days)**	13/116	11.2	44/116	37.9
**Non-consecutive (6–10 days)**	16/84	19.0	35/84	41.7

**Fig 2:**
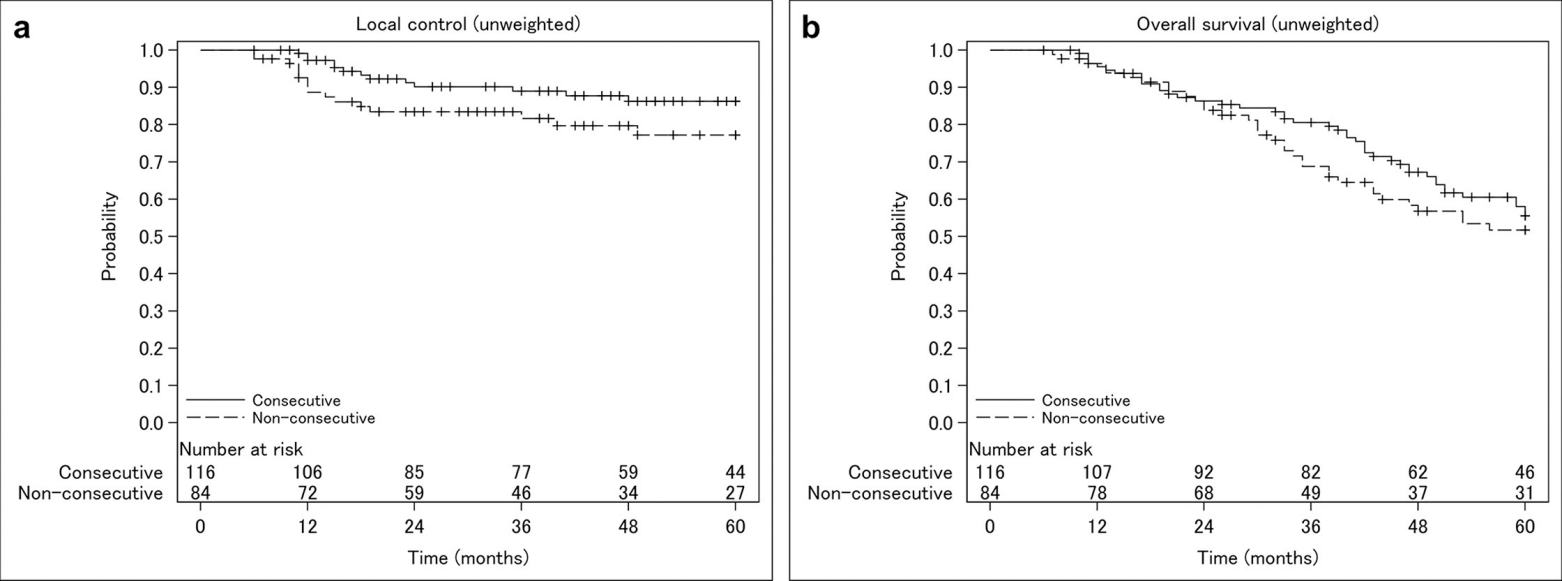
Kaplan–Meier curves of unweighted local control (a) and overall survival (b) in the treatment groups.

The unadjusted 5-year LC rate was 86.3 and 77.2% in the consecutive and non-consecutive group, respectively (log-rank test p = 0.092; HR 0.54, 95% confidence interval [CI] 0.26–1.11, p = 0.095) (Fig 2A). The unadjusted 5-year OS rate was 55.5 and 51.8% in the consecutive and non-consecutive group, respectively (log-rank test p = 0.39; HR 0.82, 95% CI 0.53–1.28, p = 0.39) (Fig 2B). After PS weighting, consecutive treatment was associated with significantly better LC (adjusted HR 0.30, 95% CI 0.14–0.65, p = 0.002) and OS (adjusted HR 0.56, 95% CI 0.34–0.91, p = 0.019) than non-consecutive treatment (S3 Fig). Furthermore, in the covariates-adjusted survival curves with PS-weighting (S3 Fig), consecutive treatment was associated with significantly better LC (adjusted HR 0.36, 95% CI 0.18–0.72, p = 0.004) and OS (adjusted HR 0.57, 95% CI 0.35–0.93, p = 0.024) than non-consecutive treatment. These results did not largely differ from that of the sensitivity analysis excluding patients with an OTT of 5 days (S1 Table).

In multivariable analyses (Table 3), the benefit of consecutive treatment on LC was confirmed across the different OTT subgroups (6 days vs. 4–5 days, HR 2.58, 95% CI 1.05–6.38, p = 0.040; 7–10 days vs. 4–5 days, HR 2.89, 95% CI 1.04–8.00, p = 0.041), while its benefit on OS was partially confirmed (6 days vs. 4–5 days, HR 2.30, 95% CI 1.29–4.10, p = 0.005; 7–10 days vs. 4–5 days, HR 1.21, 95% CI 0.61–2.38, p = 0.59).

**Table 3:** Multivariable analyses for local control and overall survival.

Covariates	Local control			Overall survival		
	HR	95% CI	p-value	HR	95% CI	p-value
**Overall treatment time (days)**						
** 6 vs. 4–5**	2.58	1.05–6.38	0.040	2.30	1.29–4.10	0.005
** 7–10 vs. 4–5**	2.89	1.04–8.00	0.041	1.21	0.61–2.38	0.59
**Age (years)**						
** 70–79 vs. 50–69**	2.15	0.44–10.6	0.35	2.02	0.81–5.09	0.13
** 80–90 vs. 50–69**	1.55	0.30–8.07	0.61	2.72	1.07–6.91	0.035
**Sex**						
** Male vs. Female**	1.52	0.44–5.19	0.51	1.67	0.80–3.48	0.17
**Histological type**						
** Squamous cell carcinoma vs. Adenocarcinoma**	1.86	0.78–4.45	0.16	1.54	0.84–2.83	0.16
** Non-biopsy-proven vs. Adenocarcinoma**	0.42	0.13–1.34	0.14	1.08	0.62–1.91	0.78
**Tumor size**						
** 1b (>2 cm, ≤3 cm) vs. 1a (≤2 cm)**	0.74	0.27–2.04	0.56	0.74	0.41–1.33	0.32
** 2a (>3 cm, ≤5 cm) vs. 1a (≤2 cm)**	1.18	0.42–3.31	0.75	1.07	0.58–1.98	0.83
**Pretreatment SUVmax**						
** >5 vs. ≤5**	3.59	1.37–9.41	0.009	2.62	1.53–4.49	<0.001
** Unknown vs. ≤5**	1.94	0.39–9.66	0.42	1.68	0.72–3.93	0.23
**Tumor location**						
** Upper/Middle lobe vs. Lower lobe**	0.87	0.38–2.01	0.75	1.09	0.67–1.79	0.72
**Charlson comorbidity index [17]**						
** >2 vs. 0–2**	1.77	0.73–4.30	0.21	2.12	1.22–3.68	0.008
**Prior history of surgery or radiotherapy for lung cancer**						
** Yes vs. No**	0.99	0.31–3.22	0.99	1.23	0.67–2.26	0.50
**Calendar period of treatment**						
** 2009–2010 vs. 2007–2008**	0.54	0.20–1.46	0.22	0.41	0.23–0.74	0.003
** 2011–2013 vs. 2007–2008**	0.80	0.29–2.19	0.67	0.49	0.26–0.91	0.025
**History of smoking**						
** Yes vs. No**	0.66	0.17–2.59	0.55	0.61	0.29–1.32	0.21

HR, hazard ratio; CI, confidence interval; SUVmax, maximum standardized uptake value.

## Discussion

This study compared the outcomes in patients with early-stage NSCLC treated with consecutive or non-consecutive SBRT and assessed the influence of the OTT with SBRT on tumor control. Our analysis showed that consecutive SBRT was associated with a marked improvement in LC compared with non-consecutive SBRT.

Our results might be explained by indirect cell death *via* tumor vascular damage, which is an emerging radiobiological concept in favor of high dose-per-fraction radiotherapy [18]. Song CW *et al*. [18, 19] proposed that a single radiation dose higher than 10 Gy induces severe vascular damage in tumors and subsequently increases tumor hypoxia, leading to the death of hypoxic cells not associated with double-stranded DNA breakage. In this context, short treatment schedules may lead to greater vascular injury and better LC than long treatment schedules. Indeed, in murine models of lung cancer, Song C *et al*. [20] reported that while the tumor vasculature collapsed 6 hours after a single irradiation dose of 15 Gy, perfusion was restored only after 2 days. Our analysis also showed that non-consecutive SBRT may be associated with poor OS. Since most patients in our cohort were diagnosed with inoperable NSCLC and could not tolerate additional treatment for local failure owing to their age and comorbidities, it may be reasonable to consider that the increased local failure in the non-consecutive group led to poor survival.

Alite *et al*. [13] reported that non-consecutive treatment (twice per week treatment, >7 days) was associated with better LC than consecutive treatment (daily treatment, ≤7 days) in patients treated with SBRT of 50 or 60 Gy in 5 fractions in a retrospective study, which is contradictory to our results. They suggested that sufficient inter-fraction intervals allowed reoxygenation of hypoxic tumor cells, resulting in improved LC. It is well known that tumor hypoxia contributes to radiotherapy resistance [21, 22]. A possible explanation for this discrepancy in results is the difference in OTT. The median OTT in the non-consecutive group was 14 days in their study, while it was only 6 days in our study. Therefore, longer inter-fraction intervals than those used in our study (i.e., OTT > 10 days) may allow for sufficient reoxygenation of the hypoxic tumor cells and contribute to tumor cell death more effectively than that by vascular injury induced using short schedule SBRT. Further research comparing outcomes between consecutive and non-consecutive treatment schedule with longer OTT than that observed in this study is needed to determine the optimal OTT.

This study had some limitations, including the retrospective study design and small sample size. We performed inverse probability of treatment weighting and covariates-adjusted analysis to adjust for differences in baseline characteristics between the treatment groups. We included the calendar period of treatment as a covariate in the analysis because both classical and modern calculation algorithms were used in this study. However, our results may be potentially affected by biases from unobserved differences. In multivariable analyses, we could not confirm the better OS observed in the consecutive group compared with that in the 7–10 days OTT subgroup. The prescribed dose used in this study was lower than what is currently used worldwide. The isocenter prescription of 48 Gy in 4 fractions was the most common dose regimen in Japan, which is similar to 42 Gy in 4 fractions (BED10 = 86.1 Gy) that cover 95% of the planning target volume [23]. The prescription dose was found to be a strong predictive factor for LC, and BED10 is currently recommended to be at least 100–105 Gy [24, 25]. Therefore, if we administer a higher dose than that used in this study cohort, the observed difference between the consecutive and non-consecutive groups might decrease.

In conclusion, prolonged OTT appears to have a negative effect on the outcomes of patients with early-stage NSCLC treated with SBRT using a short treatment schedule. However, our study, as well as previous reports, are limited by the retrospective study design and small sample size. Further research in a large population and trials that are comparative and prospective are needed to determine the optimal OTT.

## Supporting information

S1 Fig Distribution of patients according to the overall treatment time. (DOCX)

S2 Fig (a) Distribution of propensity scores between consecutive and non-consecutive treatment groups (b) Receiver operating characteristic curve discriminating the two groups (c-statistic 0.73).
(DOCX)

S3 Fig (a) Local control (a1) and overall survival (a2) curves with propensity score-weighting in the treatment groups (b) Covariates-adjusted local control (b1) and overall survival (b2) curves with propensity score-weighting in the treatment groups.
(DOCX)

S1 Table Sensitivity analysis excluding patients treated with an overall treatment time of 5 days. (DOCX)

S1 File Dataset used in this study. (CSV)
