# Supplementary figures and images for: Prolonged overall treatment time negatively affects the outcomes of stereotactic body radiotherapy for early-stage non-small-cell lung cancer: A propensity score-weighted, single-center analysis
Source: PLoS One. 2021 Jun 18;16(6):e0253203. doi: 10.1371/journal.pone.0253203 (PMC8213186; doi:10.1371/journal.pone.0253203)

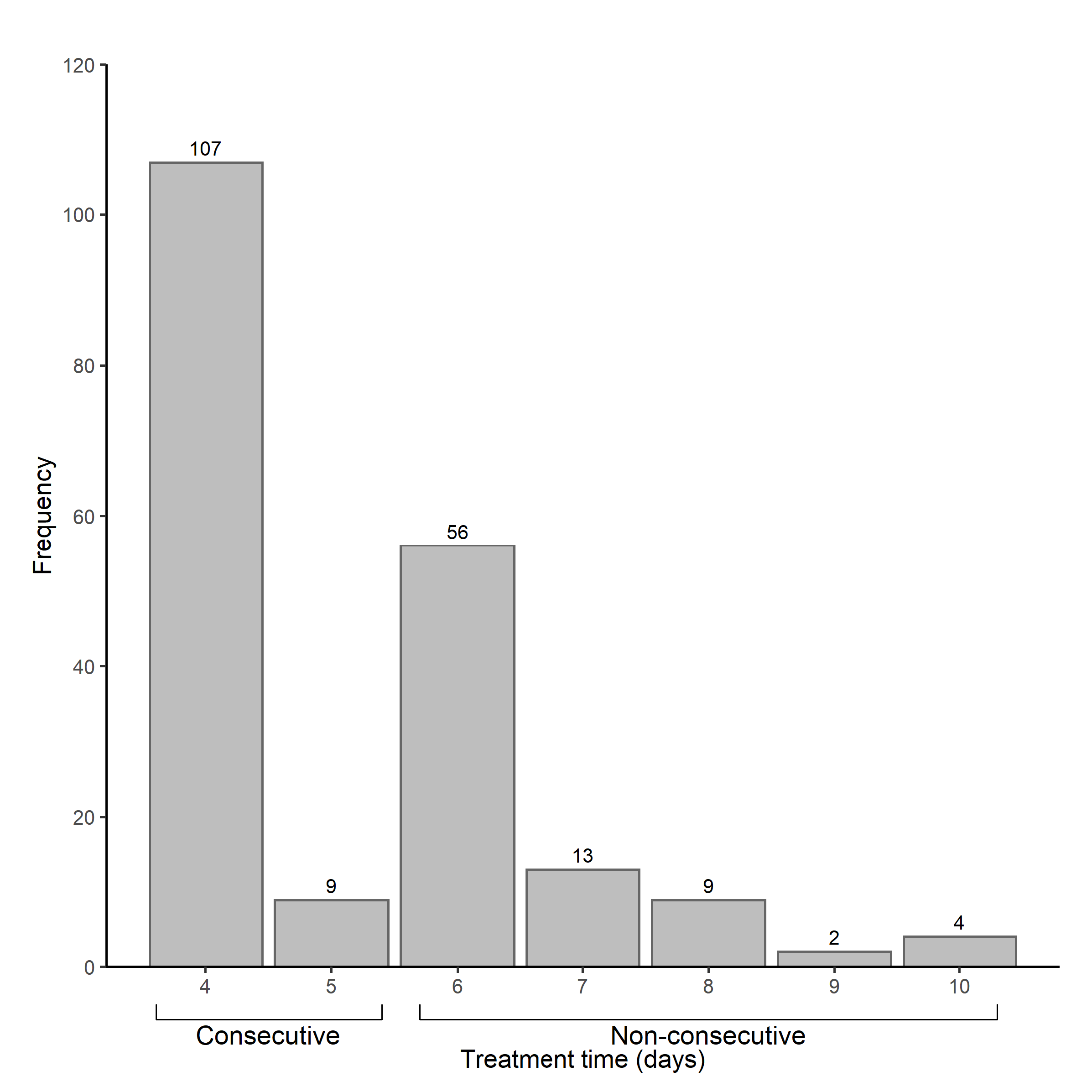


Fig. S1. Distribution of patients according to the overall treatment time

Supplement: S1 Fig — (DOCX) [file pone.0253203.s001.docx]
