# Supplementary material for: Prolonged overall treatment time negatively affects the outcomes of stereotactic body radiotherapy for early-stage non-small-cell lung cancer: A propensity score-weighted, single-center analysis
Source: PLoS One. 2021 Jun 18;16(6):e0253203. doi: 10.1371/journal.pone.0253203 (PMC8213186; doi:10.1371/journal.pone.0253203)
Supplement: S2 Fig — (a) Distribution of propensity scores between consecutive and non-consecutive treatment groups (b) Receiver operating characteristic curve discriminating the two groups (c-statistic 0.73). (DOCX) [file pone.0253203.s002.docx]

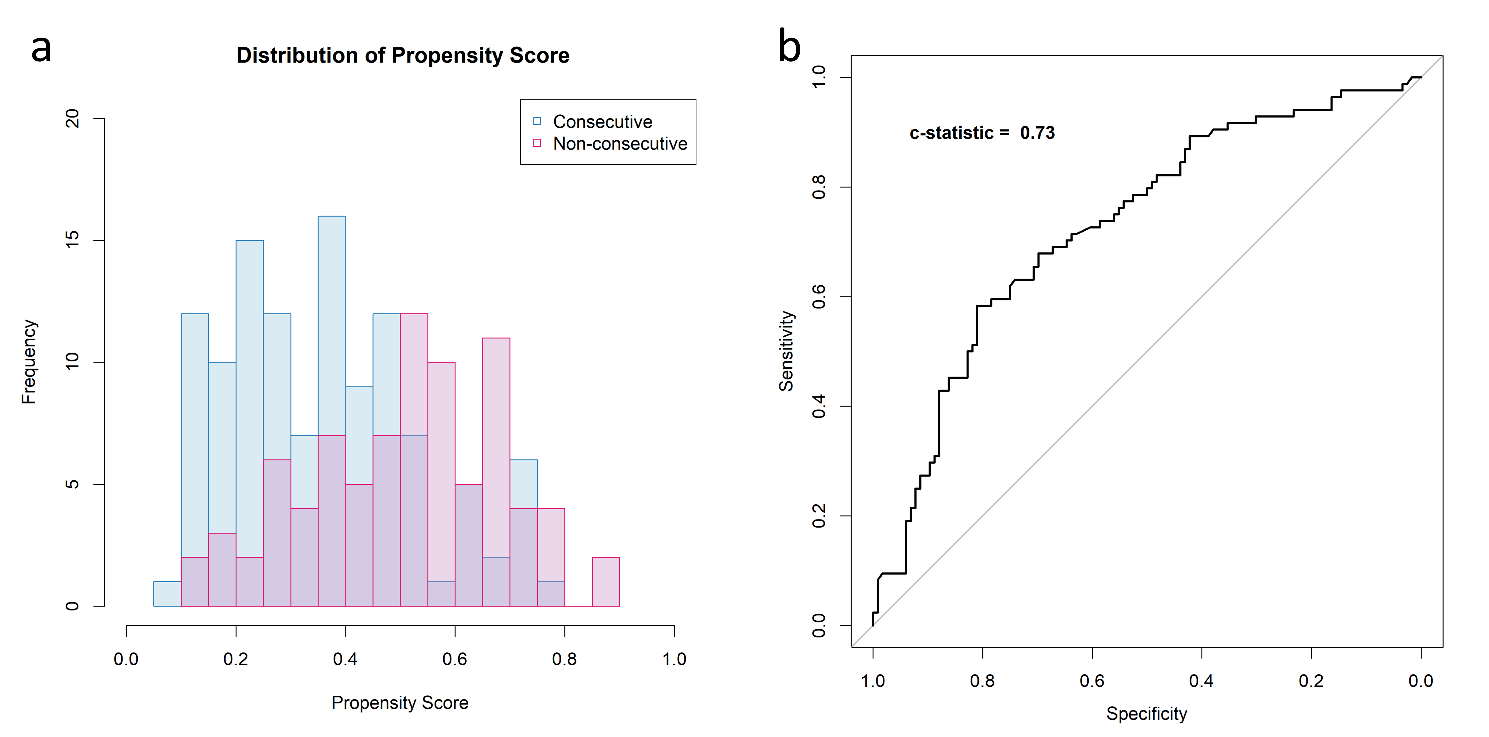


Fig. S2. (a) Distribution of propensity scores between consecutive and non-consecutive treatment groups (b) Receiver operating characteristic curve discriminating between the two groups (c-statistic 0.73)
