# Supplementary material for: Prolonged overall treatment time negatively affects the outcomes of stereotactic body radiotherapy for early-stage non-small-cell lung cancer: A propensity score-weighted, single-center analysis
Source: PLoS One. 2021 Jun 18;16(6):e0253203. doi: 10.1371/journal.pone.0253203 (PMC8213186; doi:10.1371/journal.pone.0253203)
Supplement: S3 Fig — (a) Local control (a1) and overall survival (a2) curves with propensity score-weighting in the treatment groups (b) Covariates-adjusted local control (b1) and overall survival (b2) curves with propensity score-weighting in the treatment groups. (DOCX) [file pone.0253203.s003.docx]

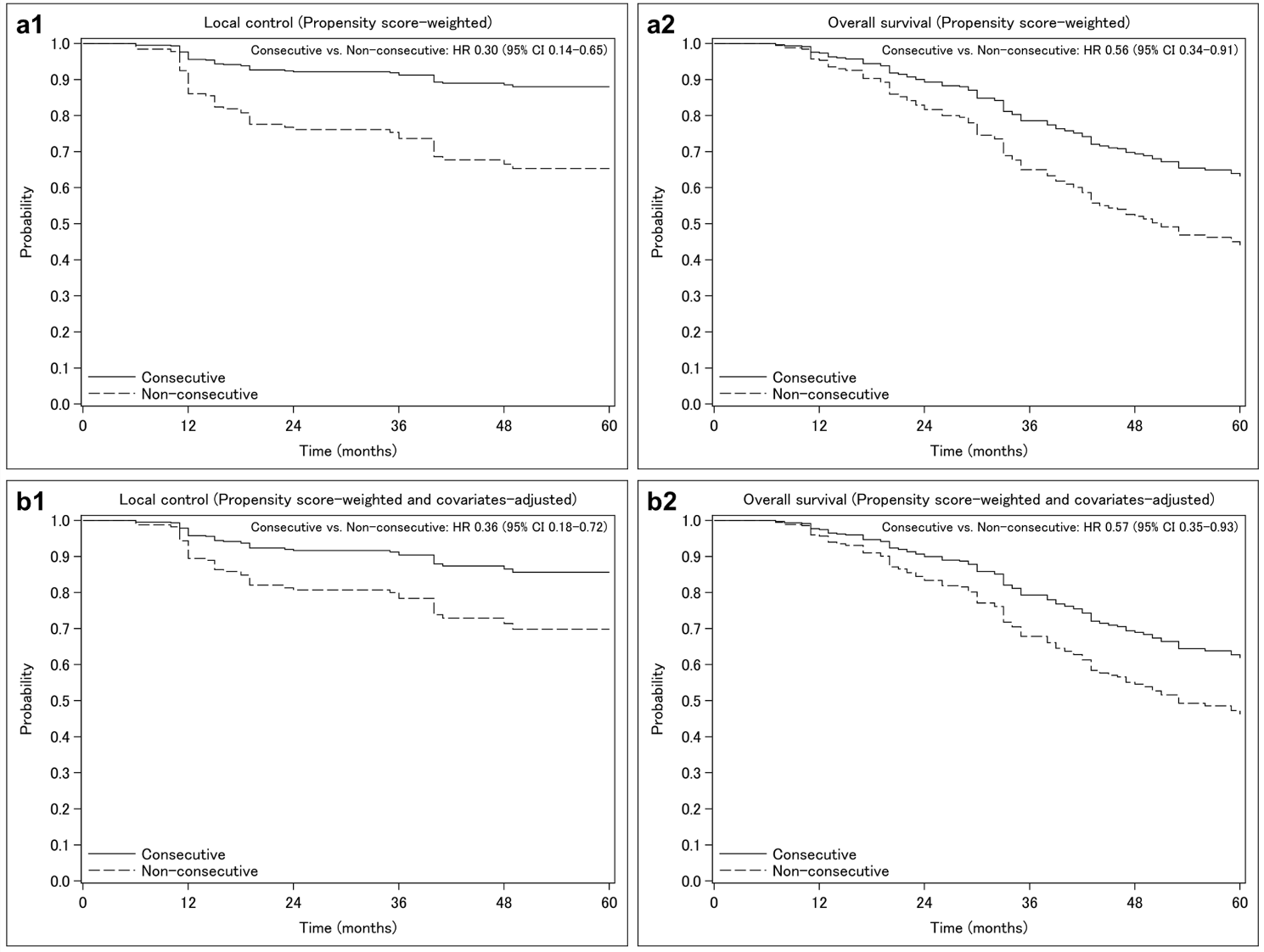
Fig. S3. (a) Local control (a1) and overall survival (a2) curves with propensity score weighting in the treatment groups (b) Covariates-adjusted local control (b1) and overall survival (b2) curves with propensity score weighting in the treatment groups
