# Supplementary material for: Prolonged overall treatment time negatively affects the outcomes of stereotactic body radiotherapy for early-stage non-small-cell lung cancer: A propensity score-weighted, single-center analysis
Source: PLoS One. 2021 Jun 18;16(6):e0253203. doi: 10.1371/journal.pone.0253203 (PMC8213186; doi:10.1371/journal.pone.0253203)
Supplement: S1 Table — (DOCX) [file pone.0253203.s004.docx]

| **S1 Table. Sensitivity analysis excluding patients treated with an overall treatment time of 5 days** | | | | |
| --- | --- | --- | --- | --- |
| **Outcome** | **HR** | **95% CI (lower)** | **95% CI (upper)** | **p-value** |
| **Local control (PS-weighted)** | 0.27 | 0.12 | 0.61 | 0.002 |
| **Local control (PS-weighted and covariates-adjusted)** | 0.32 | 0.15 | 0.69 | 0.004 |
| **Overall survival (PS-weighted)** | 0.55 | 0.34 | 0.90 | 0.017 |
| **Overall survival (PS-weighted and covariates-adjusted)** | 0.55 | 0.33 | 0.89 | 0.016 |
| PS, propensity score; HR, hazard ratio; CI, confidence interval. | | | | |
